# The microbiome of wild and mass-reared new world screwworm, *Cochliomyia hominivorax*

**DOI:** 10.1038/s41598-022-04828-5

**Published:** 2022-01-20

**Authors:** Alex P. Arp, Gladys Quintero, Agustin Sagel, Rafael Gonzales Batista, Pamela L. Phillips, Paul V. Hickner

**Affiliations:** 1 USDA-ARS, Knipling-Bushland U.S. Livestock Insect Research Laboratory, Kerrville, TX USA; 2 USDA-ARS Screwworm Research Laboratory, Pacora, Panama; 3 Panama-USA Program for the Eradication and Control of Screwworm (COPEG), Pacora, Panama; 4 USDA-APHIS-IS, Screwworm Program, Pacora, Panama

**Keywords:** Applied microbiology, Metagenomics, Entomology

## Abstract

Insect population control through continual releases of large numbers of sterile insects, called sterile insect technique (SIT), is only possible if one can mass-rear large quantities of healthy insects. Adaptation of insect stocks to rearing conditions and artificial feeding systems can have a multitude of negative effects such as inbreeding depression, reduced compatibility with wild strains, unintentional selection for traits that lower fitness after release, and an altered microbiome. Changes to insect microbiomes can have many effects on insects ranging from a reduction in sex pheromones or reduced fitness. Thus understanding these systems is important for mass rearing and the performance of the sterile insect control programs. In this study we explored the microbiome of the New World screwworm, *Cochliomyia hominivorax* (Coquerel) (Diptera: Calliphoridae) an economically important parasite of warm-blooded animals. Samples from myiases in cows and wild adults were compared to and mass-reared flies used by the SIT program. Significant differences were observed between these treatments, with wild captured flies having a significantly more diverse microbial composition. Bacteria known to stimulate oviposition were found in both wild and mass-reared flies. Two bacteria of veterinary importance were abundant in wild flies, suggesting screwworm is a potential vector of these diseases. Overall, this study provides the screwworm eradication program a platform to continue exploring the effects associated bacteria have on screwworm fitness.

## Introduction

An important role for the microbiome in the development of blow flies was demonstrated over a century ago, when it was observed that blow flies from sterilized eggs reared on sterile diet developed slower and had lower activity than flies reared in a microbial-rich environment^1^. Marion Bakula (1969) found similar results in *Drosophila*, wherein normally reared flies pupariated sooner than flies from sterilized eggs and reintroducing native bacteria to the diet of gnotobiotic flies reinvigorated fly development^2^. Additionally, she found vertical transfer of gut microbiota through the chorion, and a simple bleach and ethanol treatment could render flies axenic^2^. This method removes gut bacteria but does not prevent transfer of endosymbiotic bacteria such as *Wolbachia*, which differ in that they are vertically transferred within the egg itself and not absorbed through the chorion^3^.

Symbiotic relationships between bacteria and insect hosts are often highly specialized and the result of a long history of coevolution^4^. One of the major benefits provided by the microbiota is nutrition for the host by producing vitamins, amino acids, and fatty acids, either absent from the diet or incapable of production by the host^5^. Studies on gene expression in gnotobiotic and conventionally reared flies found that expression of digestion-associated genes in the guts of normally reared flies are increased in comparison with gnotobiotic flies, indicating further mutualism^6^. Furthermore, the modulation of gut function due to the presence of gut bacteria is tied to immune system function^7^.

Given the vital role bacteria play in the fitness of most insects, understanding these relationships could be used to improve mass-rearing systems used for sterile insect technique (SIT). SIT is an insect control method that uses continual releases of large numbers of sterilized insects to reduce fecundity and potentially eradicate wild populations of the same species. The first, and arguably most successful, use of SIT is the eradication program for the New World screwworm, *Cochliomyia hominivorax* (Coquerel 1858) (Diptera: Calliphoridae)^8, 9^. *Cochliomyia hominivorax* is an obligate parasite of warm-blooded animals including humans. Female flies lay their eggs on the periphery of a wound, which upon hatching the larvae burrow into and begin feeding on the living animal tissue, forming a myiasis. Untreated, the myiasis can lead to secondary infections and death. Economic savings to the eradicated areas, spanning the southern US to Panama and parts of the Caribbean, is estimated to be US$1.3 billion per year^10^. Currently, *C. hominivorax* is controlled by the Panama-USA Commission for the Eradication & Control of Screwworm (COPEG), which continually produces, and conducts aerial releases of millions of sterile flies per week over the Panama-Colombia border and operates an extensive network of field surveillance and outreach staff.

In the mass rearing of *C. hominivorax*, many factors are likely to influence the associated microbiota. For example, to prevent spoilage, formaldehyde is added to the larval diet which is likely to be toxic to some of the gut microbiota and any potentially endosymbiotic bacteria. Mass-reared flies are held under stable conditions in a bio secure facility, thus exposure to bacteria from typical screwworm foraging behavior or host animals is greatly reduced. Additionally, to prevent inbreeding depressions, successful strains are stored and revived from cryopreservation, a process in which eggs are bleached, dechorionated, washed in hexane, and frozen in liquid nitrogen^11^. Chorion removal and bleaching are methods used to create strains of gnotobiotic *Drosophila*, thus these steps could hinder vertical transfer of bacteria between preserved and revived strains of *C. hominivorax*^12^.

The microbiome of the New World screwworm, *C. hominivorax,* is largely unknown. One study was conducted using culture-dependent methods, to evaluate the microbiota of wild flies and the resulting wound from the myiasis^13^. The primary bacteria found in the myiasis site itself were *Escherichia coli*, *Proteus sp.*, *Proteus mirabilis*, *Staphylococcus sp.*; while the primary bacteria in the larvae were primarily *E. coli*, *Proteus sp*., *Proteus mirabilis*, and *Staphylococcus sp.* with *Moraxella phenylpyruvica*, *Pasteurella pneumotrophyca*, and *Enterococcus faecalis* exclusive to the larvae alone. However, because this study was conducted using culture-dependent methods, it could not have detected unculturable bacteria commonly associated with insects.

A study on *Cochliomyia macellaria* (Fabricius 1775), a non-parasitic species found in overlapping ranges and often in myiases with *C. hominivorax*, also through culture-dependent methods identified only four associated bacteria: *Providencia* sp., *E. coli* O157:H7, *Enterococcus faecalis*, and *Ochrobactrum* sp.^14^. In addition to identifying associated bacteria, lines of gnotobiotic flies were reared, and then fed a diet containing the bacteria found in the colony flies. Unlike *Drosophila*, which have increased fitness in the presence of their microbiota, *C. macellaria* fitness was decreased when the associated bacteria were added back to their diets^14, 15^.

Though bacteria seemed to be detrimental to the fitness of *C. macellaria*, this may not be the case in *C. hominivorax*, as females of this species prefer to oviposit in the presence of bacterial volatile compounds associated with myiasis^16^. Additionally, in *Drosophila* larval microbial exposure influences adult feeding preference^17^. Therefore, it is possible that the host preference seen in *C. hominivorax* is a learned behavior associated with specific bacteria.

In addition to host or diet preference based on microbial associations, microbial associations can have a significant impact on conspecific interactions and mate choice, though the mechanisms are not well understood^18^. In *Ceratitis capitata* (Wiedemann 1824), sterilization with radiation decreased titers of *Klebsiella* and increased the titers of potentially pathogenic *Pseudomonas* spp., resulting in reduced longevity and lowered mating success^19^. Enriching *C. capitata* diet with original microbiota or *Klebsiella* or the increased mating success and longevity of laboratory strains^19, 20^. In honeybees, microbiomes are suggested to play a role in nest-mate identification by altering cuticular hydrocarbon profiles^21^.

Here, we investigate the microbiota of *C. hominivorax* of laboratory strains, field-collected flies, and livestock wounds with myiasis using 16S next-generation sequencing. As previously described, only one culture-dependent survey of *C. hominivorax* bacterial associations has been published which used culture-dependent methods and did not include samples from mass-reared colonies used for the SIT program. It is unknown what changes to the *C. hominivorax* bacterial community have occurred during domestication and mass-rearing, and if any changes are impacting the flies used for the SIT program. This survey was conducted to characterize the microbiome of *C. hominivorax* using culture-independent methods, explore the impact of domestication on the microbiome, and provide a basis for future studies characterizing the biological importance of bacteria in this species.

## Results

### Dataset overview

Samples used in this survey were from field-collected (wild) and SIT production strain (J06) *C. hominivorax*. Wild samples were collected near Jaque, Panama, in September 2019 by COPEG staff during normal field operations. J06 samples were collected over three subsequent generations from the COPEG SIT production facility in Pacora, Panama between August and September 2019. Adult and larval samples had gut dissected and sequenced separately from body tissues. Larval environment (Diet) was also sampled from DNA extractions of artificial diet material for J06 and by swabs of the myiasis for wild samples (Table 1).

**Table 1:** Overview of samples used for the analysis of *C. hominivorax* microbiomes.

Life stage	Type	Tissue	# Samples
Adult	J06	Body	6
		Gut	6
	Wild	Body	3
		Gut	4
Larva	J06	Body	4
		Gut	6
		Diet	6
	Wild	Body	6
		Gut	5
		Myiasis Swabs	4

Two samples of J06 larval gut did not amplify during sequencing prep and one wild adult body did not pass DNA extraction and were excluded from analysis. In total 28 J06 and 22 wild *C. hominivorax* 16S rRNA gene (16S) paired-end Illumina gene libraries were produced. A total of 5,855,435 reads were obtained from 50 samples after import and quality filtering using DADA2. The mean read frequency per sample was 117,108.7 (min = 93,522.0; max = 141,124.0). Clustering resulted in 3648 amplicon sequence variants (ASVs). Taxonomic assignment of ASVs identified two kingdoms, 32 phyla, 74 classes, 132 orders, 208 families, 364 genera, and 193 species.

### Diversity comparisons

**Figure 1:**
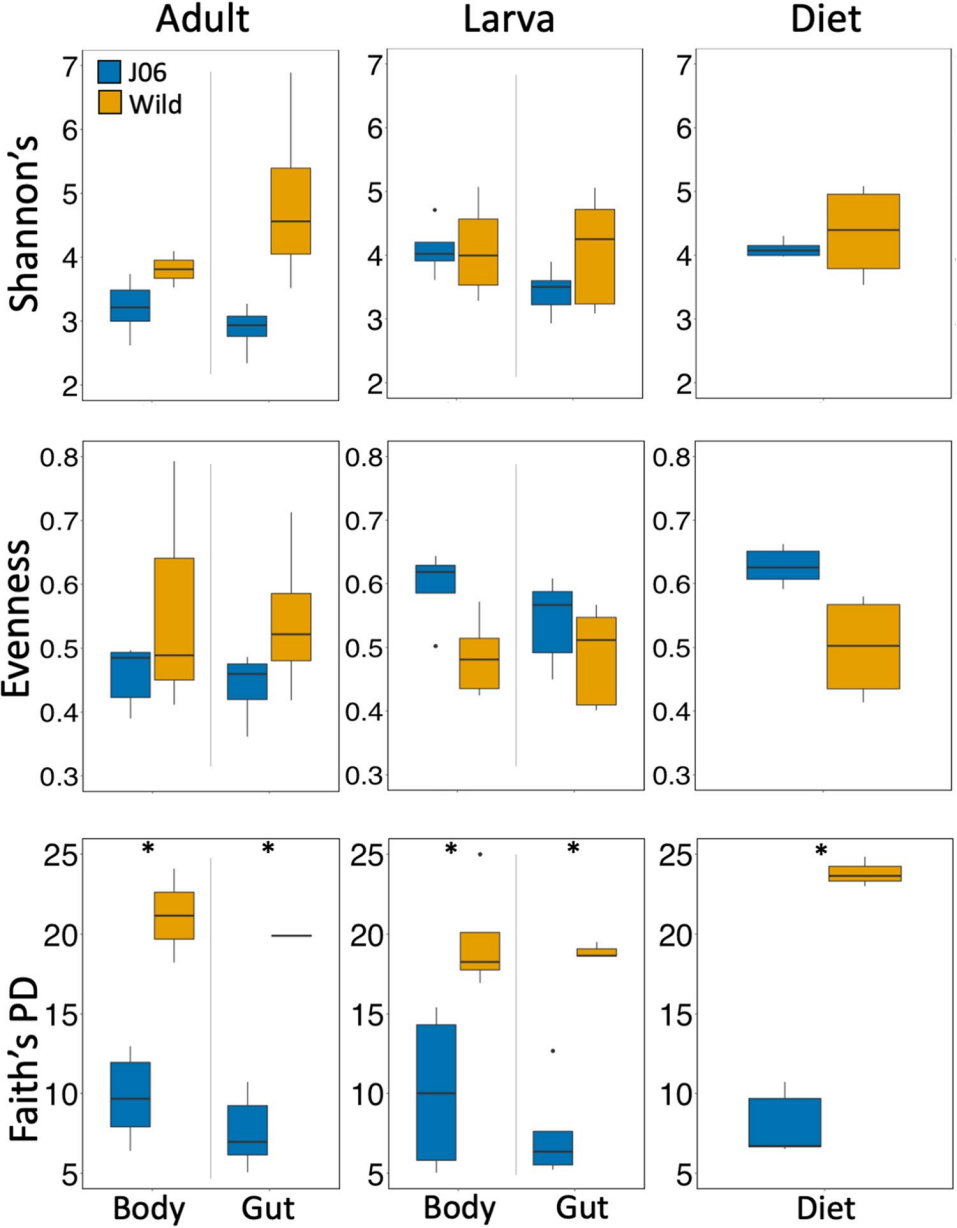
Comparisons of alpha diversity measures (Shannon’s diversity, Pielou’s evenness, and Faith’s PD) by sample type. Significant pairwise differences within sample type are indicated with asterisks.

Alpha diversity metrics provide a basis to compare the levels of diversity within samples. Here we compared within sample diversity using: Shannon’s diversity index to provide an indicator of ASV count and uniformity with a sample; Pielou’s evenness to measure of how evenly distributed ASVs are within samples; and Faith’s phylogenetic diversity (PD) which weights by the phylogenetic diversity of the ASVs within a sample. Pairwise comparisons of total J06 to wild samples found that wild samples has significantly higher diversity than J06 based on both Shannon’s diversity index (*H* = 9.657, *p-value* = 0.001) and Faith’s PD (*H* = 36.235, *p-value* < 0.001), though no difference in Pielou’s evenness (*H* = 1.033, *p-value* = 0.310). Pairwise comparisons between J06 and wild samples within sample type and life stage were not significantly different based on Shannon’s diversity, though wild samples trended higher in all cases (Fig. 1). Faith’s PD, which weights diversity by the phylogenetic diversity of microorganisms present, was significantly higher in wild samples in all pairwise comparisons (Fig. 1).

Beta diversity metrics reveal how similar the microbial communities are between sample groups and provides a basis for exploring what factors may be influencing microbial community composition. In this survey beta diversity was compared using a generalized UniFrac metric. Adonis testing of variable significance showed significant differences in beta diversity between wild and J06 (F_1,49_ = 31.337, *p*-value < 0.001), sample type (F_4,49_ = 3.414, *p*-value < 0.001), and an interaction of both variables (F_4,49_ = 2.990, *p*-value < 0.001). Permutation multivariate analysis of variance (PERMANOVA) reported significant differences between all sample types (source/life stage/material) except for body and gut from the same source and life stage (e.g., between J06 adult body and gut samples) and between all wild larvae and diet samples (Table S1). Overall, these results indicate the microbial community of *C. hominivorax* is highly diverse and impacted by both life stage, sample origin, and tissue type.

**Figure 2:**
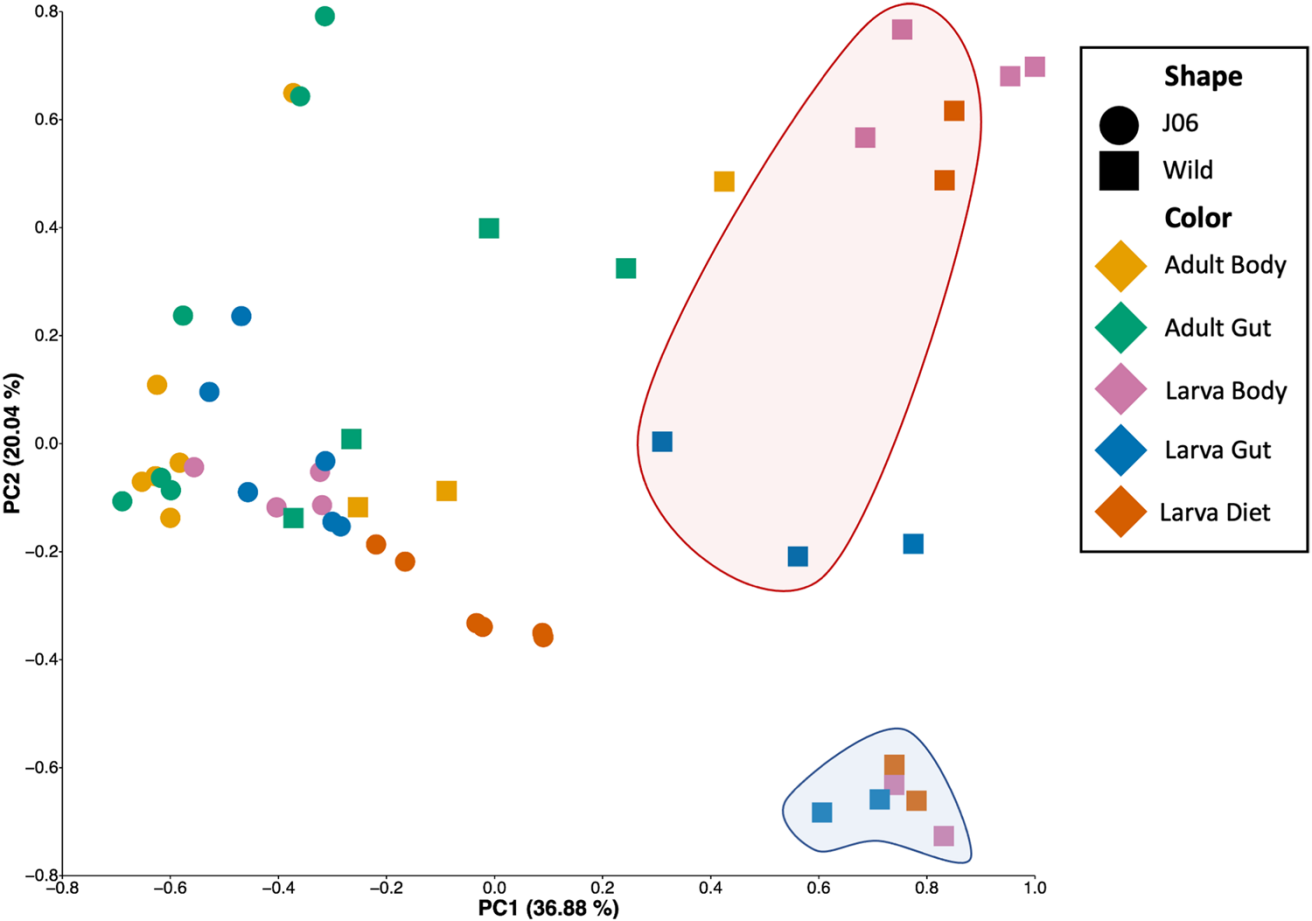
PCoA plot of generalized UniFrac distances. Blue (Myiasis 1, calf leg) and red (Myiasis 2, adult cow ear) circled samples were collected from two cows approximately 8.7 km apart, highlighting the possible microbial diversity that can occur between myiases and the impact on the larval microbiome.

Principal coordinate of analysis (PCoA) showed that samples grouped moderately by sample type (Fig. 2). The spread of points in wild samples were greater than J06, likely due to standard rearing conditions used to produce J06. Gut and body tissues from J06 adults and larvae clustered together. Interestingly, of samples of wild larvae collected from three separate myiases, two grouped together and one was tightly grouped and highly divergent along PC2.

### ASV similarity between sample types

**Figure 3:**
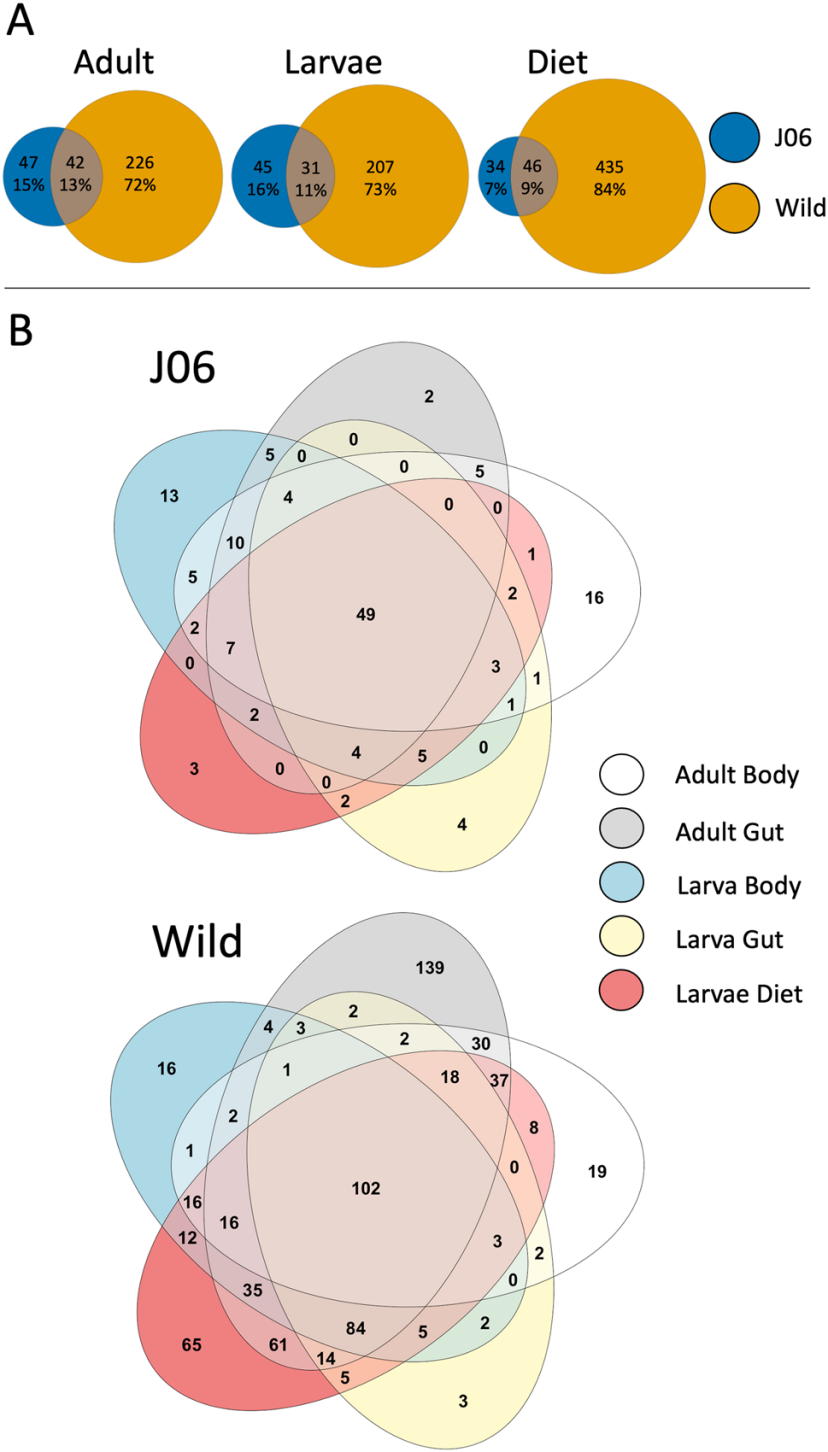
Venn Diagrams showing ASV similarity between (**A**) wild and J06 by life stage, and (**B**) sample type within J06 or wild. ASVs must have been present in half of the samples of each type to be included in the Venn diagrams.

To explore how similar the ASVs composition was between samples and sample origin, Euler plots and Venn diagrams were produced (Fig. 3). Samples from J06 show a highly reduced number of ASVs in comparison with wild samples. Close to half of the ASVs in J06 were also found in wild samples. Interestingly, these shared ASVs were highly abundant in J06 but lower in relative abundance in wild samples. In comparisons of ASVs between sample types within origin, it is apparent that J06 samples share more ASVs than are unique to sample types. In wild samples, adult guts presented the most unique ASVs, while myiasis swabs (wild larval diet) had the most abundant ASVs shared with other sample types.

### Taxonomic comparisons

**Figure 4:**
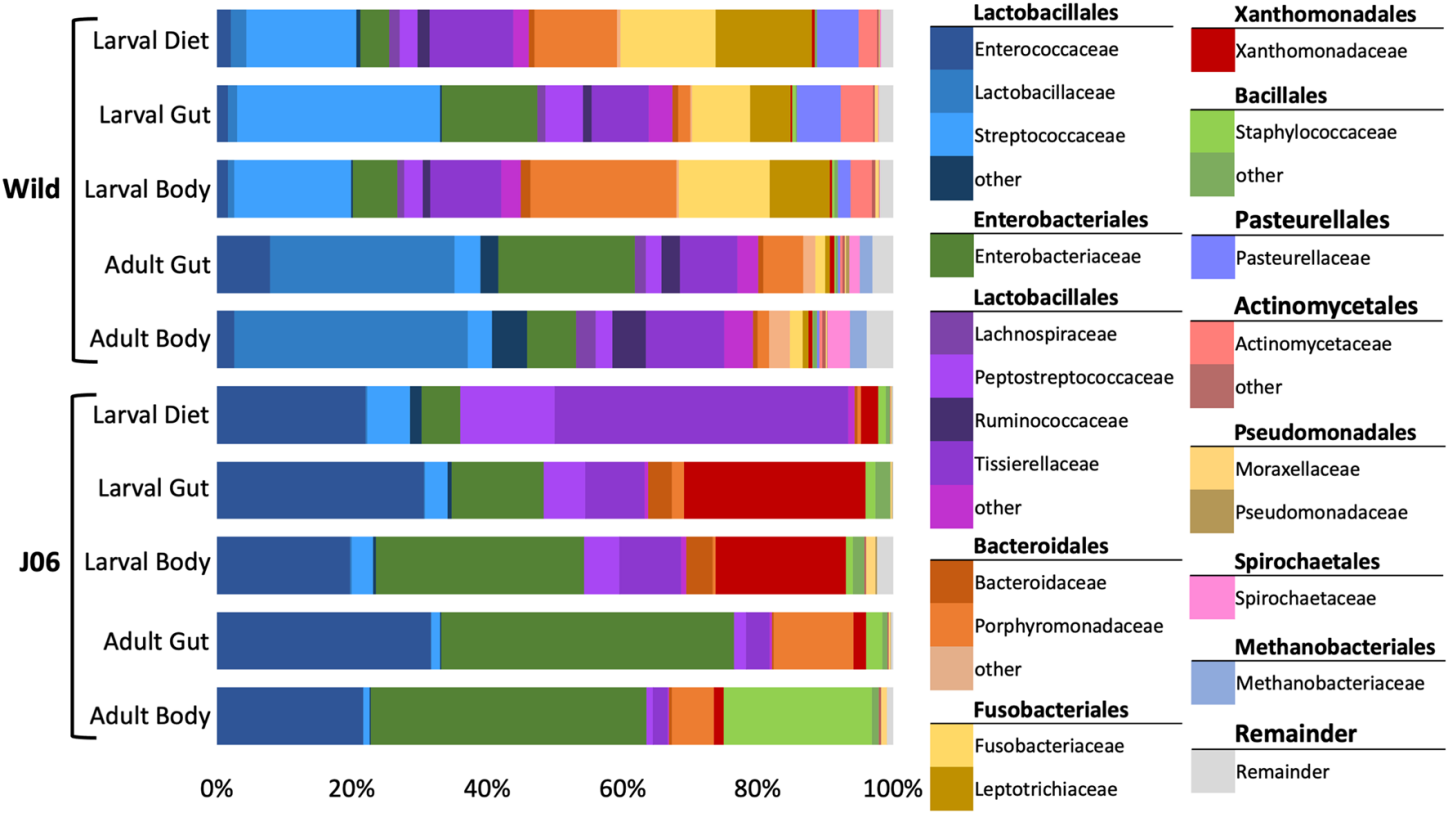
Stacked bar plots showing the composition of the average microbial community in each sample type with taxonomic classification shown to the family level. The top 12 most abundant orders and the families present within are shown.

**Figure 5:**
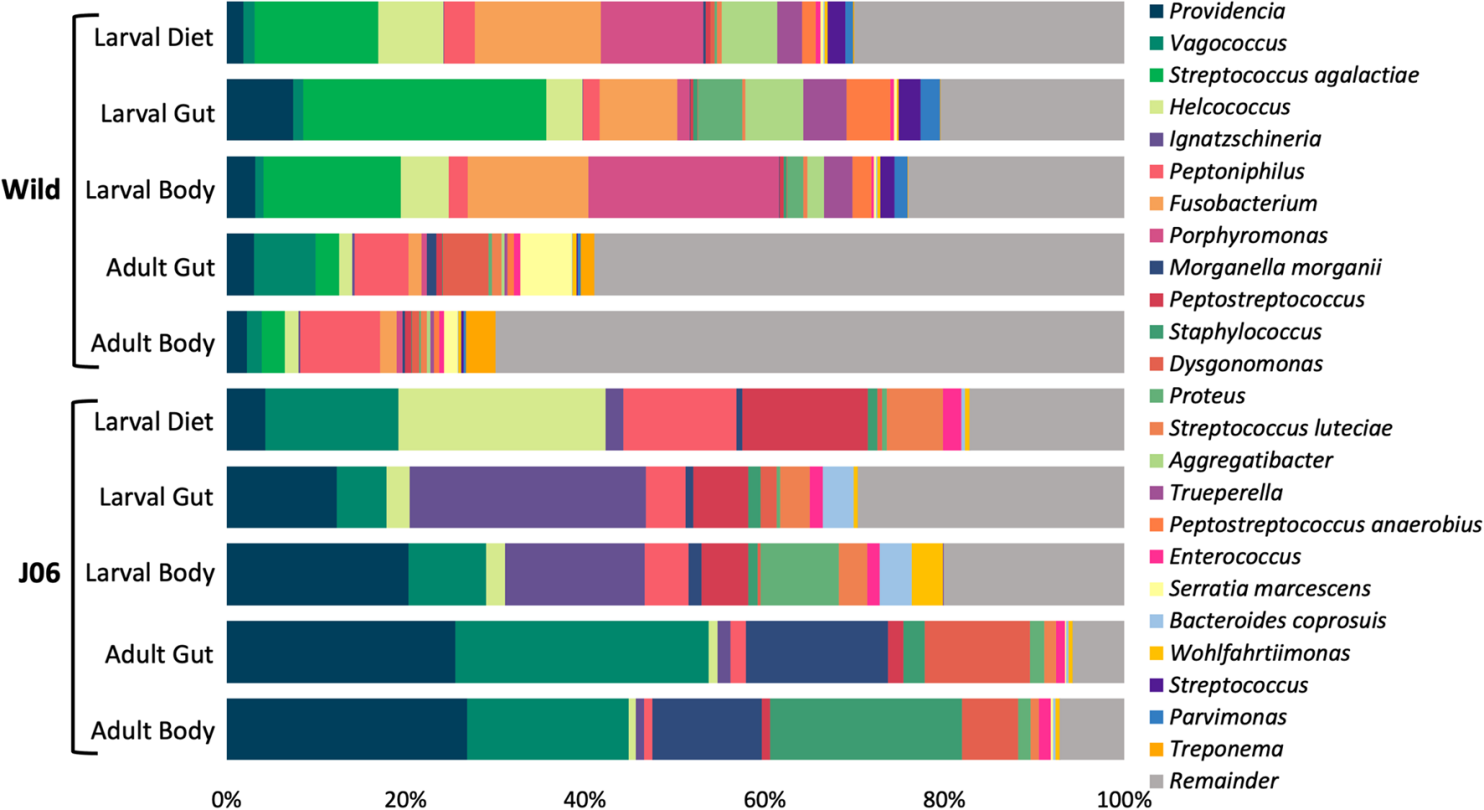
Stacked bar plot of the most abundant taxonomic classifications at the genus or species level. Bars are based on the average composition of samples within a sample type. Abundant classifications were selected by choosing taxa with a summed abundance across samples greater than 5%.

Taxonomic classification of ASVs using the Greengenes 16S rRNA database revealed large differences in the average composition of microbial communities between wild and J06 samples (Figs. 4 and 5). The most apparent difference is that J06 samples have many fewer taxa present representing a larger percentage of the composition. At the species level of classification, 70% of the wild adult body taxa each represented less than 1% of the overall composition, while in J06 over 90% of the composition was composed of 24 genera.

The most abundant families of bacteria present, reported as average percent across samples, in the J06 samples were: Enterobacteriaceae (26.82%), Enterococcaceae (25.10%), Tissierellaceae (13.39%), Xanthomonadaceae (10.37%), and Peptostreptococcaceae (5.60%). More narrowly classified to the genus level, the most abundant bacteria present were: Providencia (17.83%), Vagococcus (15.06%), Ignatzschineria (9.26%), Morganella (6.19%), and Helcococcus (5.89%).

Wild samples were dissimilar to J06 with the most abundant families consisting of: Streptococcaceae (14.22%), Lactobacillaceae (13.26%), Enterobacteriaceae (10.48%), Tissierellaceae (10.29%), and Porphyromonadaceae (8.61%). This trend followed to the genus level were the most abundant taxa were: Streptococcus (14.19%), Fusobacterium (7.88%), Porphyromonas (7.05%), Peptoniphilus (4.46%), and Helcococcus (3.92%).

Across all sample types from wild and J06 flies, the following genera were present and could represent a core microbiome or symbiotic associations: *Providencia* (10.69%), *Vagococcus* (8.71%), *Peptoniphilus* (4.68%), *Helococcus* (4.90%), *Proteus* (2.03%), and *Enterococcus* (0.95%).

Some taxa were uniquely abundant in certain sample groups and likely represent transient exposures related to environment or rearing conditions. In wild larval samples *Streptococcus agalactiae* (21.24%, mean body and gut, 13.78% diet), *Fusobacterium* (11.06%, mean body and gut; 14.02%, diet), and *Porphyromonas* (21.19%, body; 11.45%, diet) were more abundant than other sample types. Larvae of J06 had elevated levels of Ignatzschineria (20.98%, mean body and gut; 1.99%, diet) and *Peptostreptococcus* (5.67%, mean body and gut; 13.99% diet). J06 adult samples had higher levels of *Morganella morganii* (13.98%, mean body and gut) and *Staphylococcus* (21.41%, body).

**Figure 6:**
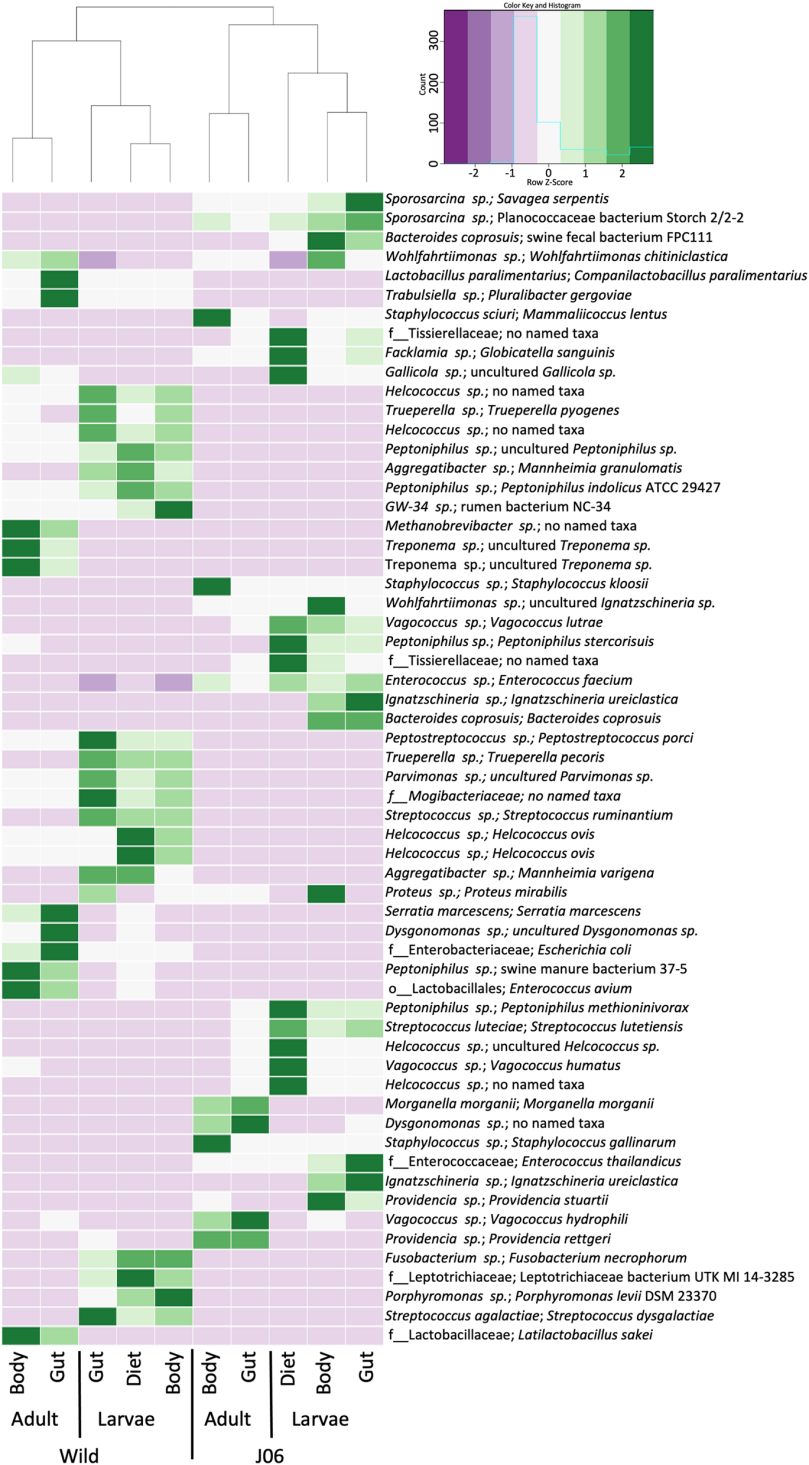
A heatmap showing the relative quantity of the top 20 most abundant ASVs per sample type. After removing duplicates, 60 unique ASVs remained. Purple represents lower abundance, while dark green is higher. The taxonomic classifications for each ASV are shown to the right side of each row, with the Greengenes classification first and the NCBI BLAST result second following the “;”. The dendrogram above the heatmap shows relative similarity based on these 60 ASVs.

To verify taxonomic classification, the topmost abundant ASVs were subjected to identification using BLAST search on the nr database. Though it should be mentioned this method can provide false positives, particularly at higher taxonomic levels, and due to limitations of the 16S V4 region sequenced. Selecting the top 20 most abundant ASVs from each sample type resulted in a list of 60 ASVs, of which the BLAST search concurred with or was more specific for 39 ASVs, disagreed with 10, and provided higher level taxonomic classification for 11 ASVs in comparison with the Greengenes (Fig. 6). Of the 11 ASVs with no taxonomic information though BLAST search, 3 were only classified to the family level by Greengenes.

## Discussion

The microbial associations of insects are vast and can have drastic impacts on behavior, nutrition, reproduction, or socialization. Identifying the composition of microbes in a species can provide the platform to further explore what impact associated bacteria can have on a host. In this study we conducted the first culture-independent microbial survey of *C. hominivorax* at different life stages, and in flies from the mass-rearing facility producing flies for the SIT program as well as wild captured flies from naturally occurring myiases. As expected, there were significant differences in the composition of microbes between wild and J06 flies, as well as an increased diversity of bacteria in wild flies. There were also differences between life stages with treatment groups, which has been observed in fruit flies and mosquitoes^22, 23^.

The microbial ecology of *C. hominivorax* was first explored using culture-based methods to identify bacteria obtained from myiasis wound swabs, larvae, and adults from purposefully infested sheep in Costa Rica^13^. In this study 43 species of bacteria were identified and the most abundant taxa (*Bacillus sp.*, *Enterococcus sp.*, *Proteus sp.*, *Providencia sp.*, *Streptococcus sp.*, and *Staphyloccus sp.*) were also abundant in our survey. Interestingly, more than half of the species identified by Caballero et al. were not present in our samples. The similar taxa identified are possibly representative of the core microbiota common to *C. hominivorax*, though a more wide-ranging survey from more locations would need to be conducted to see if this holds true across all populations. Additionally, the large difference in the additional bacteria present in the Caballero et al. (1996) survey and this survey could be a result of the animal host microbiota, influence from environmental infection, microbiota of other insects visiting the myiasis, or testing methodology.

The reduced diversity of bacteria in J06 is likely the result of the highly controlled rearing conditions within the production rearing facility at COPEG. Larval diet contains formaldehyde inhibiting microbial growth, follows strict recipes, and is discarded every generation reducing horizontal transfer between larvae^24^. Adult flies are held in a separate room and exposure to any outside species is prevented. In drastic contrast to the rearing facility, wild flies have significantly more pathways to microbial interactions. Larvae develop in wounds on animals and are exposed to the animal’s environment, as well as the bacteria present on the animals. The impact of larval environment was apparent in wild larvae collected from myiases of three different cows in a relatively close area which formed two distant clusters in the PCoA plot (Fig. 2). There is also the possibility of bacterial transfer from other insects that may be attracted to the myiasis, either to lay their own eggs or feed on the necrotic animal tissue^25^. Adult *C. hominivorax* obtain nutrients from flower nectar, animal wounds, and have been observed feeding on animal manure and may spread encountered bacteria to the wound prior to oviposition^26–28^. The microbiome of wild adult flies in this study had high abundances of *Treponema sp.*, *Methanobrevibacter sp.*, and *Lactobacillus sp.*, all of which are abundant in cow rumen and manure, which suggests that these flies had visited and possibly fed on cow excrement^29^.

One of the major challenges associated with control programs that rely on the release of sterile or genetically modified insects is the rate at which they mate with wild conspecifics; specifically, the ability of mass-reared males to outcompete their wild counterparts for females^30^. In *D. melanogaster*, assortative mating occurred between flies with different microbiota^31^. In mass-reared *C. capitata*, manipulation of the microbiota increased male sexual performance^19^. Moreover, enhancement of diet of mass-reared fruit flies with bacteria^20^ or probiotics^32^ improved performance of irradiated flies. Although the reasons for the differences in mate competition are not well understood, studies have shown that the microbiome can influence chemical communication by altering the production of pheromones^33^. One of the most striking examples is the beetle *Costelytra zealandica* which relies on *M. morganii* to produce its sex pheromone, phenol^34^. Phenol is a component of *C. hominivorax* attractant Swormlure-4, and contributes to oviposition stimulation^16, 35^. J06 adults had a high abundance of *M. morganii*, though further studies are needed to identify if the microbiome is impacting screwworm mating in *C. hominivorax*.

Mechanical, or indirect, vectoring of pathogens through physical contact, feeding, or excretions on a host animal has been studied in many flies including *Musca domestic*, *Stomoxys*, and *Chrysomya megacephala*^36, 37^. Mechanical transmission of pathogens by flies is of particular concern in livestock production where a single fly can encounter many host animals in a small area increasing transmission efficiency. In this survey, *Helcococcus sp.*, considered an emerging pathogen in livestock, was prevalent in the wild larvae and wound swabs (diet). Additionally, another livestock pathogen of economic importance, *Trueperella pyogenes,* and commonly co-occurring *Peptonophillus sp*., were identified to be in abundance in wound swabs and larvae, and in lower abundance in adult wild flies^38^. The presence of these bacteria in the wild fly samples could simply be related to typical bovine infections, though it is important to note their presence as these infections can lead to death if the animal is only treated for the myiasis with no long-term care for the wound or antibiotics. It is also reassuring that the J06 samples did not have an abundance of any known livestock pathogens and do not present a source of infections at the time of release.

Screwworm oviposition site selection is influenced by volatiles produced by wound bacteria^39^. When mixed with blood, five species of bacteria (*Klebsiella oxytoca*, *Proteus mirabilis*, *Proteus vulgaris*, *Providencia rettgeri*, and *Providencia stuartii*) produce attractive volatiles, with *P. rettgeri* being the most attractive, *K. oxytoca* the least attractive, and a combination of all five bacteria being the most attractive to gravid females^16, 39^. Two abundant ASVs were identified in all sample groups were classified these as *P. rettgeri and P. stuartii.* One abundant ASV was identified as *P. mirablis*, though no *P. vulgaris* was present, it is possible both were merged due to sequence similarity. No *Klebsiella* was identified in any samples.

It is estimated that > 40% of all insect species harbor the reproductive parasite *Wolbachia. Wolbachia* can have a variety of impacts on host reproduction, including feminization of males, sex-biased offspring, and cytoplasmic incompatibility^3^. Among the most relevant impacts to population control programs is cytoplasmic incompatibility, which results in embryonic death and is induced when a *Wolbachia*-infected fly mates with an uninfected fly, though in some species this only occurs unidirectionally in infected males mating uninfected females^40^. Utilizing cytoplasmic incompatibility as an insect control method has been successfully tested in mosquitos, either alone called incompatible insect technique (IIT), or in combination with traditional SIT (IIT-SIT) to prevent accidental introductions of fertile females carrying *Wolbachia*^41, 42^. *C. hominivorax* in this study did not have *Wolbachia* opening the possibility to utilizing artificial infections with *Wolbachia* as an alternative to radiation in the SIT program if a male-only release system was created. Transgenic systems exploiting the cytoplasmic incompatibility mechanism have also been tested and could be an additional alternative route to creating incompatible males in the absence of natural *Wolbachia* infections^43^.

*Cochliomyia hominivorax* is a major pest of livestock, wild animals, and humans, that was eradicated from North and Central America using an extensive SIT program. Continued protection of eradicated areas relies on the constant production of high-quality flies that can compete with wild populations for mates. Microbial ecology can play a major role in fly fitness and mate selection, thus we sought out to identify differences in the microbial communities of mass-reared flies used for the SIT program with wild flies. Although significant differences were identified, further studies are needed to explore the results of selective changes to the microbiome.

## Methods

### Sample collection and DNA isolation

Wild NWS flies and larvae were obtained near Jaqué, Darién Province, Panamá in September of 2019. Wild larvae NWS samples were obtained from cows and identified by COPEG staff and veterinarians performing monitoring and control of cases as part of the typical NWS barrier zone control operations approved by the Panamanian Ministry of Agriculture following approved animal handling protocols . Wild Adult NWS were captured with a hand net using traps baited with raw beef liver (purchased locally from a supermarket) aged for approximately seven days. Adult samples were placed in 15 ml cyrotubes/cryovials containing Zymo RNA/DNA shield. Larvae were collected from untreated myiases, rinsed with sterile water, and placed in tubes with RNA/DNA shield. Swabs of myiasis were collected with Zymo DNA/RNA Shield Collection Tube w/ Swab (R1106). All samples were held on ice during transportation back to the laboratory. Samples were then stored at − 20 °C.

NWS from the production strain used by the sterile insect control program at COPEG (J06) were obtained over approximately three subsequent generations, with 21 days between collection of each adult and larval replicate. Adult flies and larvae were briefly rinsed in 5% bleach solution and then 70% ethanol before storage in 70% ethanol at − 20 °C. Third instar larvae were first rinsed with sterile water then surface sterilized and stored under the same conditions as the adults. Larval diet was collected from the same trays as the larvae, placed in 15 ml cryotubes, and stored at − 20 °C.

Tissue dissections were conducted prior to DNA isolation using sterilized petri dishes for each replicate. Gut tissues were removed from adults and larvae then rinsed with sterile water prior to DNA isolation. Body tissues for larvae were the remainder of body tissues minus gut for larvae, and abdomen minus the gut for adults. Samples of J06 were pools of three gut or bodies. Wild adult samples were individual, while larvae were pools from three individuals.

DNA isolation was conducted using the ZymoBiomics DNA extraction kit according to manufacturer protocols (Zymo Research, Irvine, CA, USA). After DNA isolation, samples were checked for DNA concentration and purity using a NanoDrop 1000. Samples that did not meet quality or concentration for sequencing were purified using ethanol precipitation.

### Sequencing

Library preparation, sequencing, and quality control were completed by Novogene Corporation Inc. (Sacramento, CA, USA). The 16SV4 region of the 16S rRNA gene was amplified using 16S V4: 515F-806R primers^44^, with attached barcode. All PCR reactions were carried out with Phusion® High-Fidelity PCR Master Mix (New England Biolabs, Ipswich, Massachusetts, USA). Amplification was verified by mixing an equal volume of 1X loading buffer (containing SYBR green) with PCR product and run on a 2% agarose gel electrophoresis. Samples with bright main bands between 400 and 450 bp were chosen for further experiments. PCR product was mixed in equal ratios, then purified with Qiagen Gel Extraction Kit (Qiagen, Düsseldorf, Germany). Sequencing libraries were generated using NEBNext ® Ultra DNA Library Pre Kit for Illumina (New England Biolabs, Ipswich, Massachusetts, USA) following manufacturer's recommendations with index codes added. The library quality was assessed on the Qubit@ 2.0 Fluorometer (Thermo Scientific, Waltham, MA, USA) and Agilent Bioanalyzer 2100 system (Aligent, Santa Clara, CA, USA). Lastly, the library was sequenced on an Illumina NovaSeq 6000 platform (Illumina, Inc., San Diego, CA, USA) generating approximately 100,00 250 bp paired-end reads per sample.

Paired-end reads were assigned to samples based on their unique barcode and then truncated by cutting off the barcode and primer sequence. Paired-end reads were merged using FLASH^45^, a very fast and accurate analysis tool, which was designed to merge paired-end reads when at least some of the reads overlap, creating raw tags from reads generated from the opposite end of the same DNA fragment. Quality filtering on the raw tags was performed under specific filtering conditions to obtain the high-quality clean tags according to the QIIME (V1.7.0, http://qiime.org/index.html)^46^ quality control process^47^. The tags were compared with the reference database (Gold database, http://drive5.com/uchime/uchime_download.html) using UCHIME algorithm^48^ to detect chimeric sequences, and then the chimeric sequences were removed producing effective tags used for downstream analysis^49^. Raw sequences are available at NCBI Sequence Read Library BioProjectID: PRJNA758387.

### Analysis

Microbiome sequences were analyzed using the QIIME 2 (Ver. 2020.2)^50^ microbiome analysis package. Default settings were used unless specified. Analysis was performed on paired-end reads that were imported and demultiplexed with q2-tools-import. Amplicon sequence variant (ASV) clustering, feature table creation, and quality filtering were done with DADA 2 by q2-dada2-denoise-paired^51^. A phylogeny of ASV variants was built by aligning the sequences with MAFFT^52^ and constructed with FastTree 2^53^ using q2-align-to-tree-mafft-fasttree. Taxonomic classification of ASVs was conducted with comparison to the Greengenes 99% OTU database v13.8^54^ with the q2-feature-classifier^55^. Secondary identification of ASVs was conducted on the average top 20 most abundant ASVs in each sample type. ASV sequences were extracted and submitted to identification by the National Center for Biotechnology Information (NCBI) BLASTn suite against the rn/nt database. The highest similarity sequence with species level classification was used for comparison. The heatmap showing ASV abundance and comparison of Greengenes classification to BLAST result was created in R with the heatmap.2 function of the gplots (ver. 3.1.1) package.

Core statistical data was analyzed with q2-diversity-core-metrics-phylogenetics using a sampling depth of 30,000 reads, based on alpha rarefaction curves for each sample (Fig. S1). Alpha diversity metrics Shannon’s diversity, Pielou's evenness, and Faith’s PD^56^ figures were created by importing the Qiime2 statistics data into R^57^ using the qiime2R package (https://github.com/jbisanz/qiime2R) and plotting using ggplot (https://ggplot2.tidyverse.org). Pairwise comparisons of alpha and beta diversities between wild and production samples were calculated with q2-diversity-alpha-group-significance using the Kruskal–Wallis test.

Beta diversity comparisons were calculated using a generalized UniFrac^58^ distance metric using q2-diversity-beta-phylogenetic with an alpha of 0.5. Pairwise comparisons of sample type and tissue were analyzed with q2-diversity-beta-group-significance using permutational multivariate analysis of variance (PERMANOVA). Significance of beta diversity variable interactions were evaluated using a multiway ADONIS test with q2-diversity-adonis. PCoA using generalized UniFrac distances was used to determine the similarities of microbial communities between sample type, life stage or tissue. The PCoA plot was created using a sampling depth of 30,000 reads and 100 iterations and plotted with Vega (https://vega.github.io/vega/; Ver. 5.17.0).

Venn diagrams and Euler plots of ASV similarity were created using the MicEco R package (https://github.com/Russel88/MicEco; Ver. 0.9.16) with the minimum ASV presence set to 50% of samples.

## Supplementary Information

Supplementary Information.

## References

[1] Wollmann E (1911). Sur l’e ´levage des mouches ste ´riles. Contribution a‘ la con- naissance du roˆ le des microbes dans les voies digestive. Ann. Inst. Pasteu.

[2] Bakula M (1969). The persistence of a microbial flora during postembryogenesis of *Drosophila melanogaster*. J. Invertebr. Pathol..

[3] Kaur R, Shropshire JD, Cross KL, Leigh B, Mansueto AJ, Stewart V, Bordenstein SR, Bordenstein SR (2021). Living in the endosymbiotic world of *Wolbachia*: A centennial review. Cell Host Microbe.

[4] Douglas AE (2011). Lessons from studying insect symbioses. Cell Host Microbe.

[5] Engel P, Moran NA (2013). The gut microbiota of insects: Diversity in structure and function. FEMS Microbiol. Rev..

[6] Combe BE, Defaye A, Bozonnet N, Puthier D, Royet J, Leulier F (2014). *Drosophila* microbiota modulates host metabolic gene expression via IMD/NF-κB signaling. PLoS One.

[7] Broderick NA, Buchon N, Lemaitre B (2014). Microbiota-induced changes in *Drosophila*
*melanogaster* host gene expression and gut morphology. MBio.

[8] Knipling EF (1957). Control of screw-worm fly by atomic radiation. Sci. Mon..

[9] Scott MJ, Concha C, Welch JB, Phillips PL, Skoda SR (2017). Review of research advances in the screwworm eradication program over the past 25 years. Entomol. Exp. Appl..

[10] Vargas-Terán M, Hofmann HC, Tweddle NE, Dyck VA, Hendrichs J, Robinson AS (2005). Impact of screwworm eradication programmes using the sterile insect technique. Sterile Insect Technique: Principles and Practice in Area-Wide Integrated Pest Management.

[11] Leopold RA, Wang WB, Berkebile DR, Freeman TP (2001). Cryopreservation of embryos of the new world screwworm *Cochliomyia hominivorax* (Diptera: Calliphoridae). Ann. Entomol. Soc. Am..

[12] Koyle ML, Veloz M, Judd AM, Wong ACN, Newell PD, Douglas AE, Chaston JM (2016). Rearing the fruit fly *Drosophila melanogaster* under axenic and gnotobiotic conditions. J. Vis. Exp..

[13] Caballero M, Hernández G, Poudevigne F, Ruiz-Martínez I (1996). Isolation and identification of bacteria associated with the screwworm fly *Cochliomyia hominivorax*, (Coquerel) and its myiasis. Ann. N. Y. Acad. Sci..

[14] Ahmad A, Broce A, Zurek L (2006). Evaluation of significance of bacteria in larval development of *Cochliomyia macellaria* (Diptera: Calliphoridae). J. Med. Entomol..

[15] Keebaugh ES, Yamada R, Obadia B, Ludington WB, Ja WW (2018). Microbial quantity impacts *Drosophila* nutrition, development, and lifespan. iScience.

[16] Zhu JJ, Chaudhury MF, Durso LM, Sagel A, Skoda SR, Jelvez-Serra NS, Santanab EG (2017). Semiochemicals released from five bacteria identified from animal wounds infested by primary screwworms and their effects on fly behavioral activity. PLoS ONE.

[17] Wong ACN, Wang QP, Morimoto J, Senior AM, Lihoreau M, Neely GG, Simpson SJ, Ponton F (2017). Gut microbiota modifies olfactory-guided microbial preferences and foraging decisions in *Drosophila*. Curr. Biol..

[18] Engl T, Michalkova V, Weiss BL, Uzel GD, Takac P, Miller WJ, Abd-Alla AMM, Aksoy S, Kaltenpoth M (2018). Effect of antibiotic treatment and gamma-irradiation on cuticular hydrocarbon profiles and mate choice in tsetse flies (*Glossina m. morsitans*). BMC Microbiol..

[19] Ami E, Ben; Yuval, B., Jurkevitch, E.  (2010). Manipulation of the microbiota of mass-reared Mediterranean fruit flies *Ceratitis capitata* (Diptera: Tephritidae) improves sterile male sexual performance. ISME J..

[20] Gavriel S, Jurkevitch E, Gazit Y, Yuval B (2011). Bacterially enriched diet improves sexual performance of sterile male Mediterranean fruit flies. J. Appl. Entomol..

[21] Vernier CL, Chin IM, Adu-Oppong B, Krupp JJ, Levine J, Dantas G, Ben-Shahar Y (2020). The gut microbiome defines social group membership in honey bee colonies. Sci. Adv..

[22] Taylor PW, Sutcliffe B, Chapman TA, Majumder R (2020). Microbiome of the queensland fruit fly through metamorphosis. Microorganisms.

[23] Wang Y, Gilbreath TM, Kukutla P, Yan G, Xu J (2011). Dynamic gut microbiome across life history of the malaria mosquito *Anopheles gambiae* in Kenya. PLoS ONE.

[24] Thomas JK, Fadul GJ, Keller GP, Chaudhury MF (2018). The use of dried bovine hemoglobin and plasma for mass rearing New World screwworm. J. Insect Sci..

[25] Hall MJR, Wall RL, Stevens JR (2016). Traumatic myiasis: A neglected disease in a changing world. Annu. Rev. Entomol..

[26] Mackley JW, Snow JW (1982). Effects of cattle dung on the behavior and survival of adult screwworms and on the development of their eggs. Environ. Entomol..

[27] Thomas DB, Mangan RL (1989). Oviposition and wound-visiting behavior of the screwworm fly, *Cochliomyia hominivorax* (Diptera: Calliphoridae). Ann. Entomol. Soc. Am..

[28] Mackley JW, Long GL (1983). Behavior of sterile adult screwworms (Diptera: Calliphoridae) on flowering trees and shrubs. Ann. Entomol. Soc. Am..

[29] Leahy SC, Kelly WJ, Ronimus RS, Wedlock N, Altermann E, Attwood GT (2013). Genome sequencing of rumen bacteria and archaea and its application to methane mitigation strategies. Animal.

[30] Sutter A, Price TA, Wedell N (2021). The impact of female mating strategies on the success of insect control technologies. Curr. Opin. Insect Sci..

[31] Sharon G, Segal D, Ringo JM, Hefetz A, Zilber-Rosenberg I, Rosenberg E (2010). Commensal bacteria play a role in mating preference of *Drosophila melanogaster*. Proc. Natl. Acad. Sci. U. S. A..

[32] Cai Z, Yao Z, Li Y, Xi Z, Bourtzis K, Zhao Z, Bai S, Zhang H (2018). Intestinal probiotics restore the ecological fitness decline of *Bactrocera dorsalis* by irradiation. Evol. Appl..

[33] Engl T, Kaltenpoth M (2018). Influence of microbial symbionts on insect pheromones. Nat. Prod. Rep..

[34] Marshall DG, Jackson TA, Unelius CR, Wee SL, Young SD, Townsend RJ, Suckling DM (2016). *Morganella morganii* bacteria produces phenol as the sex pheromone of the New Zealand grass grub from tyrosine in the colleterial gland. Sci. Nat..

[35] Mackley JW, Brown HE (1984). Swormlure-4: A new formulation of the Swormlure-2 mixture as an attractant for adult screwworms, *Cochliomyia hominivorax* (Diptera: Calliphoridae). J. Econ. Entomol..

[36] Baldacchino F. *et al.* Transmission of pathogens by *Stomoxys* flies (Diptera, Muscidae): A review. *Parasite*. *20* (2013) 10.1051/parasite/2013026 PMC3756335 23985165

[37] Issa R (2019). *Musca domestica* acts as transport vector hosts. Bull. Natl. Res. Cent.

[38] Rzewuska M, Kwiecień E, Chrobak-Chmiel D, Kizerwetter-Świda M, Stefańska I, Gieryńska M (2019). Pathogenicity and virulence of *Trueperella pyogenes*: A review. Int. J. Mol. Sci.

[39] Chaudhury MF, Zhu JJ, Skoda SR (2016). Bacterial volatiles attract gravid secondary screwworms (Diptera: Calliphoridae). J. Econ. Entomol..

[40] Zabalou S, Riegler M, Theodorakopoulou M, Stauffer C, Savakis C, Bourtzis K (2004). *Wolbachia*-induced cytoplasmic incompatibility as a means for insect pest population control. Proc. Natl. Acad. Sci. U. S. A..

[41] Crawford JE, Clarke DW, Criswell V, Desnoyer M, Cornel D, Deegan B, Gong K, Hopkins KC, Howell P, Hyde JS (2018). Efficient production of male *Wolbachia*-infected *Aedes aegypti* mosquitoes enables large-scale suppression of wild populations. Nat. Biotechnol..

[42] Zheng X, Zhang D, Li Y, Yang C, Wu Y, Liang X, Liang Y, Pan X, Hu L, Sun Q (2019). Incompatible and sterile insect techniques combined eliminate mosquitoes. Nature.

[43] Le Page DP, Metcalf JA, Bordenstein SR, On J, Perlmutter JI, Shropshire JD, Layton EM, Funkhouser-Jones LJ, Beckmann JF, Bordenstein SR (2017). Prophage WO genes recapitulate and enhance *Wolbachia*-induced cytoplasmic incompatibility. Nature.

[44] Walters W, Hyde ER, Berg-Lyons D, Ackermann G, Humphrey G, Parada A, Gilbert JA, Jansson JK, Caporaso JG, Fuhrman JA (2016). Improved bacterial 16S rRNA gene and fungal internal transcribed spacer marker gene (V4 and V4-5) primers for microbial community surveys. mSystems.

[45] Magoč T, Salzberg SL (2011). FLASH: Fast length adjustment of short reads to improve genome assemblies. Bioinformatics.

[46] Caporaso JG, Kuczynski J, Stombaugh J, Bittinger K, Bushman FD, Costello EK, Fierer N, Peña AG, Goodrich JK, Gordon JI (2010). correspondence QIIME allows analysis of high- throughput community sequencing data Intensity normalization improves color calling in SOLiD sequencing. Nat. Publ. Gr..

[47] Bokulich NA, Subramanian S, Faith JJ, Gevers D, Gordon JI, Knight R, Mills DA, Caporaso JG (2013). Quality-filtering vastly improves diversity estimates from Illumina amplicon sequencing. Nat. Methods.

[48] Edgar RC, Haas BJ, Clemente JC, Quince C, Knight R (2011). UCHIME improves sensitivity and speed of chimera detection. Bioinformatics.

[49] Haas BJ, Gevers D, Earl AM, Feldgarden M, Ward DV, Giannoukos G, Ciulla D, Tabbaa D, Highlander SK, Sodergren E (2011). Chimeric 16S rRNA sequence formation and detection in Sanger and 454-pyrosequenced PCR amplicons. Genome Res..

[50] Bolyen E, Rideout JR, Dillon MR, Bokulich NA, Abnet CC, Al-Ghalith GA, Alexander H, Alm EJ, Arumugam M, Asnicar F (2019). Reproducible, interactive, scalable and extensible microbiome data science using QIIME 2. Nat. Biotechnol..

[51] Callahan BJ, McMurdie PJ, Rosen MJ, Han AW, Johnson AJA, Holmes SP (2016). DADA2: High-resolution sample inference from Illumina amplicon data. Nat. Methods.

[52] Katoh K, Misawa K, Kuma KI, Miyata T (2002). MAFFT: A novel method for rapid multiple sequence alignment based on fast Fourier transform. Nucleic Acids Res..

[53] Price MN, Dehal PS, Arkin AP (2010). FastTree 2: Approximately maximum-likelihood trees for large alignments. PLoS One.

[54] McDonald D, Price MN, Goodrich J, Nawrocki EP, Desantis TZ, Probst A, Andersen GL, Knight R, Hugenholtz P (2012). An improved Greengenes taxonomy with explicit ranks for ecological and evolutionary analyses of bacteria and archaea. ISME J..

[55] Bokulich NA, Kaehler BD, Rideout JR, Dillon M, Bolyen E, Knight R, Huttley GA, Gregory Caporaso J (2018). Optimizing taxonomic classification of marker-gene amplicon sequences with QIIME 2’s q2-feature-classifier plugin. Microbiome.

[56] Faith DP (1992). Conservation evaluation and phylogenetic diversity. Biol. Conserv..

[57] Team, R. C. R: A language and environment for statistical computing. *R Found. Stat. Comput.* (2016).

[58] Chen J, Bittinger K, Charlson ES, Hoffmann C, Lewis J, Wu GD, Collman RG, Bushman FD, Li H (2012). Associating microbiome composition with environmental covariates using generalized UniFrac distances. Bioinformatics.

