# Supplementary material for: The microbiome of wild and mass-reared new world screwworm, Cochliomyia hominivorax
Source: Sci Rep. 2022 Jan 20;12:1042. doi: 10.1038/s41598-022-04828-5 (PMC8776964; doi:10.1038/s41598-022-04828-5)
Supplement: Supplementary file 1 — Supplementary Information. [file 41598_2022_4828_MOESM1_ESM.docx]

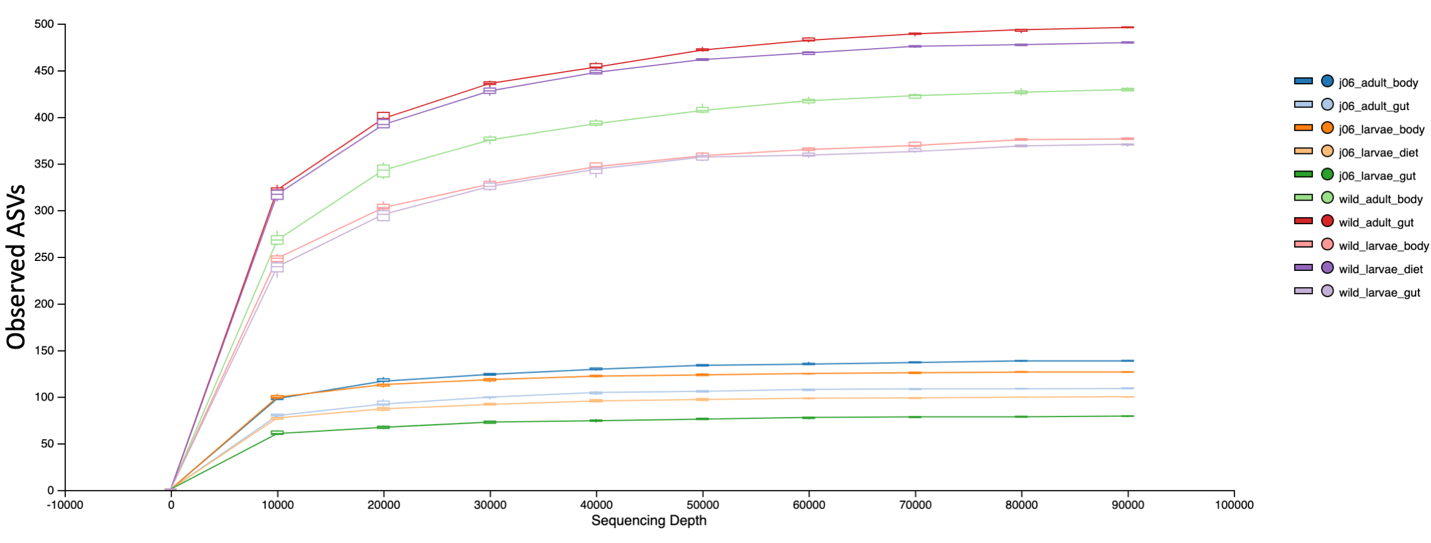


Figure S1. Alpha rarefaction plot showing the observed ASVs at sampling depths 0 to 90,000 at 10,000 read intervals.

Table S1. Pairwise significance values of PERMANOVA beta-diversity between sample and tissue types.

| **Group 1** | **Group 2** | **Sample size** | **pseudo-F** | **p-value** | **q-value** | **Significant** |
| --- | --- | --- | --- | --- | --- | --- |
| j06_adult_body | j06_adult_gut | 12 | 1.001 | 0.448 | 0.458 |  |
|  | j06_larvae_body | 10 | 3.733 | 0.006 | 0.012 | * |
|  | j06_larvae_diet | 12 | 14.644 | 0.001 | 0.010 | * |
|  | j06_larvae_gut | 12 | 5.770 | 0.004 | 0.011 | * |
|  | wild_adult_body | 9 | 6.161 | 0.014 | 0.020 | * |
|  | wild_adult_gut | 10 | 5.758 | 0.003 | 0.010 | * |
|  | wild_larvae_body | 12 | 10.985 | 0.003 | 0.010 | * |
|  | wild_larvae_diet | 10 | 12.192 | 0.004 | 0.011 | * |
|  | wild_larvae_gut | 11 | 9.389 | 0.003 | 0.010 | * |
| j06_adult_gut | j06_larvae_body | 10 | 3.579 | 0.006 | 0.012 | * |
|  | j06_larvae_diet | 12 | 13.866 | 0.005 | 0.012 | * |
|  | j06_larvae_gut | 12 | 4.961 | 0.002 | 0.010 | * |
|  | wild_adult_body | 9 | 6.616 | 0.014 | 0.020 | * |
|  | wild_adult_gut | 10 | 5.973 | 0.01 | 0.017 | * |
|  | wild_larvae_body | 12 | 10.867 | 0.003 | 0.010 | * |
|  | wild_larvae_diet | 10 | 12.612 | 0.002 | 0.010 | * |
|  | wild_larvae_gut | 11 | 10.100 | 0.002 | 0.010 | * |
| j06_larvae_body | j06_larvae_diet | 10 | 7.202 | 0.011 | 0.018 | * |
|  | j06_larvae_gut | 10 | 1.719 | 0.081 | 0.091 |  |
|  | wild_adult_body | 7 | 3.781 | 0.034 | 0.041 | * |
|  | wild_adult_gut | 8 | 3.646 | 0.035 | 0.041 | * |
|  | wild_larvae_body | 10 | 7.118 | 0.004 | 0.011 | * |
|  | wild_larvae_diet | 8 | 7.733 | 0.025 | 0.033 | * |
|  | wild_larvae_gut | 9 | 5.770 | 0.006 | 0.012 | * |
| j06_larvae_diet | j06_larvae_gut | 12 | 8.681 | 0.003 | 0.010 | * |
|  | wild_adult_body | 9 | 8.877 | 0.013 | 0.020 | * |
|  | wild_adult_gut | 10 | 9.418 | 0.01 | 0.017 | * |
|  | wild_larvae_body | 12 | 13.702 | 0.003 | 0.010 | * |
|  | wild_larvae_diet | 10 | 17.253 | 0.006 | 0.012 | * |
|  | wild_larvae_gut | 11 | 12.511 | 0.001 | 0.010 | * |
| j06_larvae_gut | wild_adult_body | 9 | 6.249 | 0.011 | 0.018 | * |
|  | wild_adult_gut | 10 | 6.099 | 0.003 | 0.010 | * |
|  | wild_larvae_body | 12 | 11.186 | 0.004 | 0.011 | * |
|  | wild_larvae_diet | 10 | 12.843 | 0.003 | 0.010 | * |
|  | wild_larvae_gut | 11 | 9.786 | 0.006 | 0.012 | * |
| wild_adult_body | wild_adult_gut | 7 | 0.304 | 0.846 | 0.846 |  |
|  | wild_larvae_body | 9 | 2.485 | 0.028 | 0.036 | * |
|  | wild_larvae_diet | 7 | 2.302 | 0.063 | 0.073 | * |
|  | wild_larvae_gut | 8 | 2.310 | 0.019 | 0.026 | * |
| wild_adult_gut | wild_larvae_body | 10 | 2.868 | 0.014 | 0.020 | * |
|  | wild_larvae_diet | 8 | 2.437 | 0.03 | 0.038 | * |
|  | wild_larvae_gut | 9 | 2.661 | 0.007 | 0.013 | * |
| wild_larvae_body | wild_larvae_diet | 10 | 0.777 | 0.393 | 0.411 |  |
|  | wild_larvae_gut | 11 | 1.418 | 0.18 | 0.198 |  |
| wild_larvae_diet | wild_larvae_gut | 9 | 1.135 | 0.313 | 0.335 |  |
